# AGE-FRIENDLY ENVIRONMENT PROMOTES WELL-BEING IN PEOPLE WITH SUBJECTIVE MEMORY IMPAIRMENT

**DOI:** 10.1093/geroni/igad104.0006

**Published:** 2023-12-21

**Authors:** Odelyah Saad

**Affiliations:** Lev Academic Center, Jerusalem, Yerushalayim, Israel

## Abstract

Objectives Older people with cognitive impairment are more likely to feel lonely and to have lower well-being compared to others (Shankar et al., 2017). One way to overcome difficulties and loneliness is to live in an Age Friendly Environment (AFE) (Gibney et al., 2020). Living in an AFE enables older people to stay active even when function deteriorates (Cramm et al., 2018) and thus- maintain low loneliness and high well-being (Domènech-Abella et al., 2021). Approximately 18%-25% of people aged 60 and over whose objective cognitive function is normal report a subjective cognitive impairment (Röhr et al., 2020; Zullo et al., 2021). They are less active (Parikh et al., 2016) and so, they are lonelier (Montejo Carrasco et al., 2020). The aim of this study was to examine whether living in an AFE enables people coping with subjective memory impairment to maintain higher well-being than those who don’t. Methods Applying a correlational study design, 105 community dwelling older adults (mean age 74.5; SD-6.54), 62% of whom were women, were recruited. Hayes’ PROCESS macro (Model 7) assessed relationships between socio-demographic data, subjective social support (SS), loneliness, a sense of belonging, AFE, active ageing and negative affect (NA). Results Findings demonstrated a significant indirect association between age-friendly environments and negative affect, which was moderated by subjective memory impairment. Moreover, we found an interaction between AFE and memory.. Those with SMI experienced less loneliness in an AFE In conclusion, AFE is crucial for individuals with SMI to reduce loneliness and negative affect. Conclusion This study is the first to show that when AFE is low, people with subjective memory impairment cope with more loneliness compared to those without it, but when AFE is high then there is no difference in the level of loneliness between people with or without subjective memory impairment. Hence, AFE is especially significant for those coping with subjective memory impairment for reducing loneliness and negative affect. Authorities should strive to create AFE to promote active aging and high WB of older people living in the community.

